# Dynamic substrate preferences predict metabolic properties of a simple microbial consortium

**DOI:** 10.1186/s12859-017-1478-2

**Published:** 2017-01-23

**Authors:** Onur Erbilgin, Benjamin P. Bowen, Suzanne M. Kosina, Stefan Jenkins, Rebecca K. Lau, Trent R. Northen

**Affiliations:** 1 Environmental Genomics and Systems Biology Division, Lawrence Berkeley National Laboratory, 1 Cyclotron Road, Berkeley, CA 94720 USA; 2 Joint Genome Institute, 2800 Mitchell Dr, Walnut Creek, CA 94598 USA; 3 Present Address: Intrexon Corporation, 1750 Kraft Dr, Blacksburg, VA 24060 USA

**Keywords:** Microbiology, Quantitative metabolomics, Substrate preferences, Predicting community function

## Abstract

**Background:**

Mixed cultures of different microbial species are increasingly being used to carry out a specific biochemical function in lieu of engineering a single microbe to do the same task. However, knowing how different species’ metabolisms will integrate to reach a desired outcome is a difficult problem that has been studied in great detail using steady-state models. However, many biotechnological processes, as well as natural habitats, represent a more dynamic system. Examining how individual species use resources in their growth medium or environment (exometabolomics) over time in batch culture conditions can provide rich phenotypic data that encompasses regulation and transporters, creating an opportunity to integrate the data into a predictive model of resource use by a mixed community.

**Results:**

Here we use exometabolomic profiling to examine the time-varying substrate depletion from a mixture of 19 amino acids and glucose by two *Pseudomonas* and one *Bacillus* species isolated from ground water. Contrary to studies in model organisms, we found surprisingly few correlations between resource preferences and maximal growth rate or biomass composition. We then modeled patterns of substrate depletion, and used these models to examine if substrate usage preferences and substrate depletion kinetics of individual isolates can be used to predict the metabolism of a co-culture of the isolates. We found that most of the substrates fit the model predictions, except for glucose and histidine, which were depleted more slowly than predicted, and proline, glycine, glutamate, lysine and arginine, which were all consumed significantly faster.

**Conclusions:**

Our results indicate that a significant portion of a model community’s overall metabolism can be predicted based on the metabolism of the individuals. Based on the nature of our model, the resources that significantly deviate from the prediction highlight potential metabolic pathways affected by species-species interactions, which when further studied can potentially be used to modulate microbial community structure and/or function.

**Electronic supplementary material:**

The online version of this article (doi:10.1186/s12859-017-1478-2) contains supplementary material, which is available to authorized users.

## Background

While some work on mixed-substrate growth has been performed in continuous culture at steady state [1], understanding substrate usage and competition in batch cultures may have both ecological and practical applications. Many environmental processes happen with pulsed inputs: for example the release of substrates into the soil following rainfall, light–dark cycles, digestion in animals, etc. Additionally, some biotechnologies that use microorganisms are also batch processes, such as the large-scale fermentations of microbe-processed foods (e.g. cheese, wine, etc.). Most of these processes use mixed microbial cultures, including one-pot processes of biomass conversion to biofuels and other biosynthetic products [2–4]. Studying the temporal substrate utilization by individuals is an important first step in developing approaches to better model these biochemical processes.

Exometabolomics, also known as metabolic footprinting, is a powerful platform for studying how microbes and their consortia modify substrate pools, as analysis is only of the extracellular metabolites [5]. With the development of exometabolomics pipelines, the metabolic connections between microbes have begun to be studied at a large scale and have allowed for a more comprehensive approach to monitoring the dynamic transformations of relatively complex mixtures of substrates [5]. Some key examples include optimizing multiple steps of lignocellulose degradation [6, 7], understanding metabolic interactions between species in mixed communities [8], and determining the ecological role of individuals within a mixed community [9–11]. We have recently found exometabolite niche partitioning in two soil environments where sympatric microbes were found to target largely non-overlapping portions of the available substrates, thus minimizing substrate competition [10]. These experiments were focused on the endpoint depletion of substrates by isolates, not the temporal sequence of utilization. However, the order of substrate utilization (i.e. substrate preferences) may further discriminate the adaptive strategies of individual organisms for common substrates.

In addition to exometabolomics, several genomics-enabled analyses have been used to model cellular metabolism and metabolic interactions between species in mixed communities [12]. However, this type of analysis relies on the availability of a sequenced genome for each organism, and for the genome to be properly annotated. No genome annotation is perfect; there are both false positives (presence of a function when there is none) and negatives (assertion that a function is missing when it is not). There is also the issue of genomes that harbor a significant number of genes of unknown or hypothetical function. These genomes may very well harbor full biochemical pathways that cannot be predicted based on our current databases. New biochemical pathways are constantly being discovered and characterized, and the presence of these novel pathways would drastically alter the reconstructed metabolic network of a species. Furthermore, while these models can be used to investigate the space of potential interspecies interactions without optimizing an objective function (e.g. biomass or ATP production), predicting which interactions actually happen in the environment require optimization to predict what the in- and out-fluxes would be, and thus how the metabolisms of different species would network together. Here we have focused on developing a model based on experimental data of how microbial species deplete resources over time, with no assumptions made based on genomic data.

As recently shown in the pioneering work by Behrends et al., the kinetics of substrate depletion from a mixture of substrates can be effectively fit using a few parameters [13]: see Eq. (1) in Methods. When compared across all substrates in an environment, these parameters have great potential in providing a direct measure of an organism’s substrate preferences within that environment, effectively creating a resource usage model for the organism. When taken into consideration with other species’ models, they may enable the prediction of the overall net metabolism of microbial consortia by aggregating individual contributions to environmental substrate usage. Observed deviations from these predictions could help identify interspecies interactions that modulate an organism’s metabolism, e.g. communication and antagonism between microbes within communities.

Here we compare the temporal depletion of 20 substrates by three isolates and fit these data to the Behrends model (Eq. 1), describing their substrate preferences within this ‘environment’. We then examine if the first substrates depleted result in maximal growth rate, or relate to growth medium or biomass composition. Finally, we developed a model that simply combines the usage profiles of individual species to test if a consortium initially composed of an equal mixture of each of the three isolates consumes substrates in an identical manner to when they are grown individually, i.e. the presence of other microbes does not affect their substrate usage. Any deviations from this model may indicate compounds that are actively regulated. For example, if a compound is consumed significantly faster or earlier than predicted by the model, this would indicate an additional interaction between species such as synergistic or competitive growth.

This modeling approach is a first step to uncovering the fundamental metabolic interactions within microbial communities. It serves as a test: if a resource behaves as modeled (passes the test), its use is not affected by the presence of other species. On the other hand, if a resource fails the test, this indicates that that particular resource may be influencing a phenotypic change in at least one species to gain a competitive advantage, or involved in a larger exometabolic network that connects different species. When this test is applied to a well-defined ecosystem, it will highlight the “important” resources in that environment, narrowing down the number of metabolic interactions to study in an environment. Furthermore, new data on how the resources are used can be incorporated as parameters into this model, improving its ability to accurately model how all of the resources are used by a community.

## Results and Discussion

In order to determine the substrate usage profiles of individuals, we designed a defined medium composed of sufficient levels of standard vitamins, minerals, phosphate, and ammonium and limiting levels of carbon (glucose and nineteen amino acids (see Methods). This medium was designed such that the species would reach stationary phase within 12 h and every substrate could be detected in a single LC-MS run.

Bacilli and pseudomonads represent some of the most ubiquitous soil bacteria, and we selected the common soil bacterium *Bacillus cereus* for comparison with two closely related *Pseudomonas* species, *Pseudomonas lini* and *Pseudomonas baetica* (Additional file 1: Figure S1) that were isolated from groundwater; taxonomic assertions were confirmed by BLAST search results on the sequenced 16S rRNA gene. For simplicity, we will refer to the species as *Bc* (*Bacillus cereus*), *Pl*, (*Pseudomonas lini*) and *Pb* (*Pseudomonas baetica*). Each species was grown individually in the defined medium, with supernatant samples collected every hour for 12 h, and one final time point at 26 h.

**Fig. 1:**
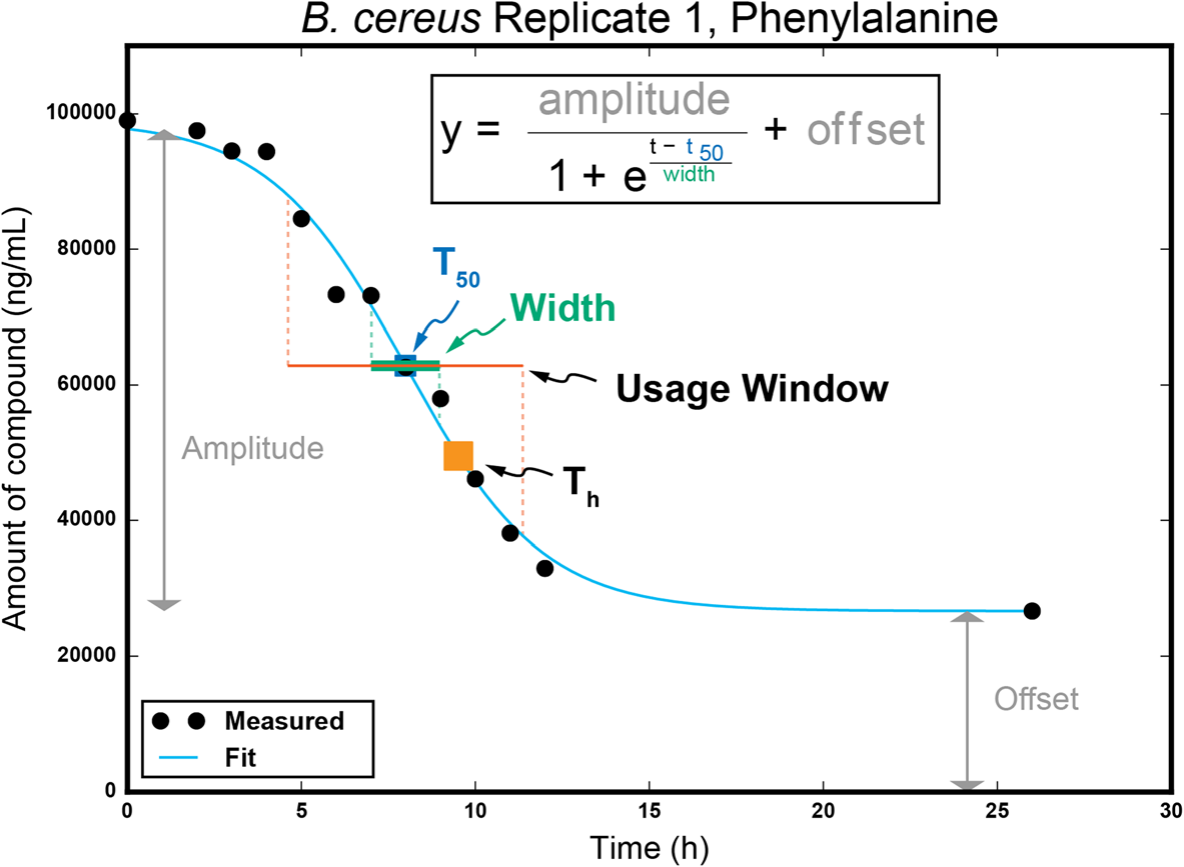
Modeling usage parameters. Example curve fitting to Behrends model (*cyan*). *Blue square* indicates the modeled T_50_ parameter of the Behrends model, or inflection point of the curve, and the width parameter of the model is depicted by the *green bar* centered at T_50_. The *orange square* represents the calculated T_h_ value, or when half of the total amount of compound has been depleted, and the *red bar* depicts the calculated usage window, or time when the compound is depleted from 90 to 10% of the total amount used by the species

The absolute concentrations of the 20 growth substrates were quantified at each time point, and the data were fit to a previously described model for compound depletion during microbial batch culture [13] (Fig. 1, Algorithm 1). We observed that all compounds followed the Behrends model over the course of growth for each species, with the exception of two compounds: glycine increased over the first 5 h of culture from all three species and then decreased logarithmically, and the methionine depletion profile for *Bc* was indeterminable due to both variance in the data and a lack of time points from 12 to 24 h (Additional file 2). Overall, these observations corroborate previous assertions that substrate utilization by microbes in batch culture follow the shape of a logistic growth type curve [13–15]. It is important to note that in our assay, the disappearance of signal does not necessarily mean that a compound is utilized by an organism. The compound may be enzymatically transformed to a different compound outside of the cell and then utilized, or it may be simply be imported into the cell and not participate in any metabolism. While strange, the latter scenario has been reported to occur in Cyanobacteria [16].

**Fig. 2:**
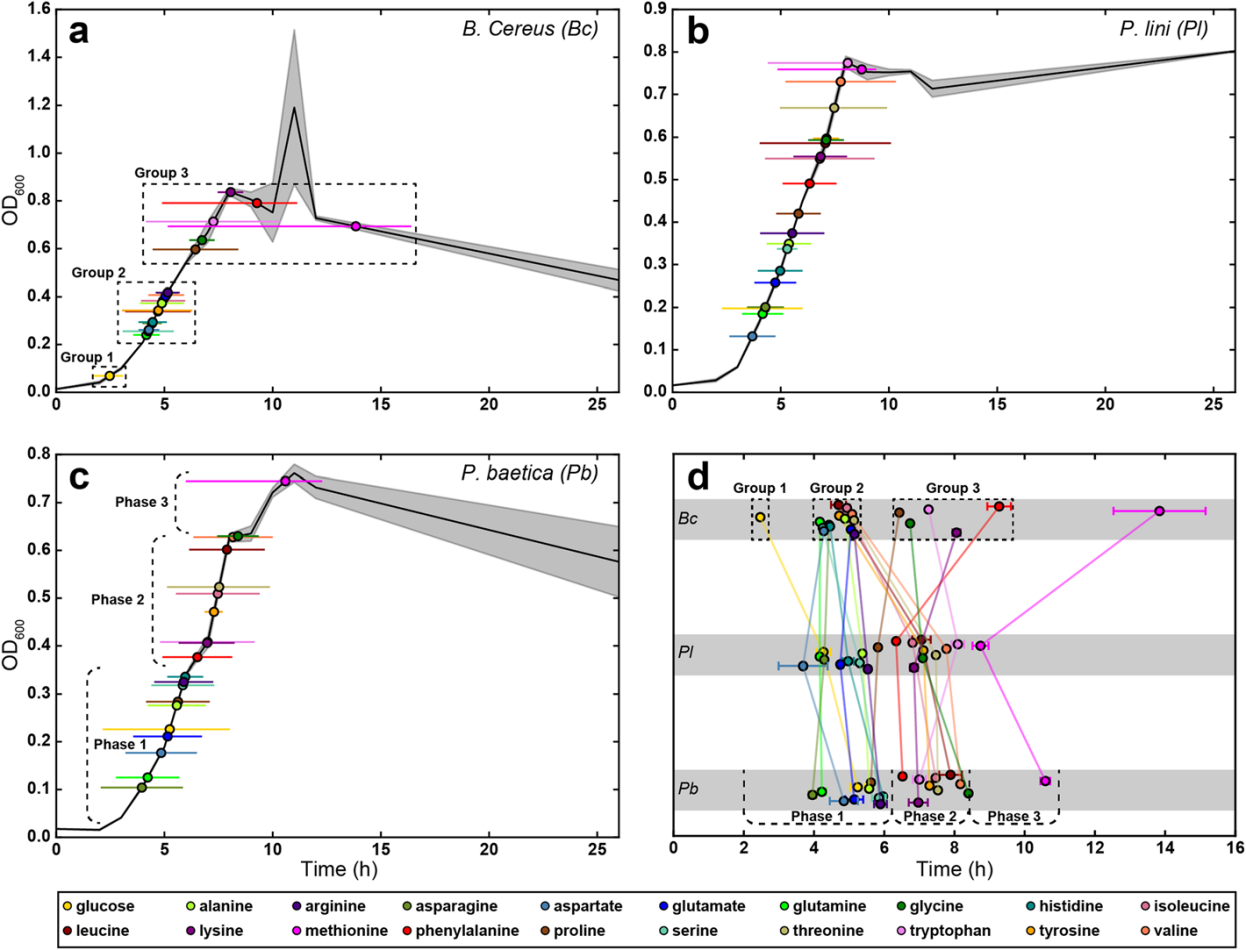
T_h_ and width values for the strains. **a**–**c** T_h_ and width for each compound mapped onto the growth curve of each strain. *Colored circles* represent average T_h_ and *colored horizontal lines* represent the average usage window (time of depletion from 90 to 10% of total resource used by the strain). *Solid black line* is the average OD_600_ of each strain measured over time (*n* = 3), with shading representing standard deviation. **d** Comparison of T_h_ values between strains, of all compounds, with *error bars* representing standard error. *Dashed boxes* in **a** and **d** indicate the grouping of compounds utilized by *Bc*, and *dashed brackets* in **c** and **d** indicate the different growth phases observed for *Pb*

To examine the sequence of substrate depletion in finer detail, we used the model to calculate the time at which each species depleted half of the total amount of each compound (T_h_), and when the compound was depleted from 90% to 10% of the total amount available to the species (usage window) (Fig. 1), and mapped them onto the growth curve of each species (Fig. 2a–c). For *Bc*, we observed that compounds were half-depleted in three distinct groups (Figs. 2a and d, dotted boxes). *Bc* initially utilized glucose, then a cluster of 13 amino acids that all had T_h_ values within 0.25 h of each other during early logarithmic growth, and finally half-depleted the remaining six substrates in late exponential and stationary phases. Neither of the pseudomonads appeared to utilize substrates in these types of groups, but instead had a more even distribution throughout their growth curve (Figs. 2b–d). However, the growth curve of *Pb* did show multiple growth phases (Fig. 2c), and so compounds can be mapped to the growth phase in which they are half-depleted (Fig. 2d). This observation is more in line with the traditional view of catabolite repression and multi-auxic growth, where a lag phase will be observed each time the organism reorganizes its metabolism to utilize different substrates [17].

It is surprising that for these three species we observed three different combinations of growth curve and substrate utilization profile: a temporally distinct grouping of compound utilization with only one observed growth phase (Fig. 2a), an even distribution of substrate utilization with only one growth phase (Fig. 2b) and an even distribution over multiple growth phases (Fig. 2c). This is quite significant given that two of the species belong to the same genus (*Pl* and *Pb*). This suggests that the metabolic regulatory systems between the two species are different: while *Pb* slows down its growth, presumably because it is undergoing a large-scale “switch” of metabolic systems, *Pl* does not, which may indicate that either all its metabolic systems are constitutively active, or the regulation of the systems is so perfectly timed that the organism can seamlessly switch from one metabolic regime to another. *Bc* may also have an efficient metabolic regulatory system, as even though we observe distinct temporal gaps between groups of compounds, we did not observe multiple growth phases.

To compare the differences in substrate depletion between species, we compared T_h_ across the three species (Fig. 2d and Additional file 3: Table S1). Across all three species, glutamine, glutamate, alanine, arginine, proline and asparagine, were half-depleted within 1 h of each other. Additionally, the T_h_ values across all substrates for the two *Pseudomonas* species were close, but not identical, consistent with their short phylogenetic distance but different species identity (Fig. 2d); a similar observation has been described previously [15]. Considering the differences in growth curves between the two species, this is quite intriguing, as the general order in which the species consume the metabolites is not different, but there is this difference in growth profiles, supporting the hypothesis that there could be significant physiological differences between such closely related species.

*Bc* was markedly different from the two pseudomonads, differing greatly in the amount of time it depleted 8 of the compounds (Fig. 2d and Additional file 3: Table S1). Of these, the utilization of glucose was particularly interesting, as it was predominantly depleted before there was any appreciable increase in biomass (Fig. 2a). This may indicate that there is a significant delay in substrate conversion to biomass in this species, or that *Bc* rapidly transforms glucose into some other compound, for example glycogen.

We next wondered if the preferred substrates offer some physiological benefit over less preferable substrates. It is a general assumption in microbiology that substrates consumed first may be more advantageous than those consumed later [18], and that this would depend on the competitive ‘strategy’ of the organism. Major strategies suggested include maximal growth rate and maximal biomass yield. Generally, copiotrophs (organisms that grow in nutrient-rich conditions) are thought of as r-strategists (maximal growth rate) and oligotrophs (organisms that can only grow in low-nutrient conditions) as K-strategists (maximum yield) [19, 20]. The strains used in this study are copiotrophs, and we would expect that their order of substrate consumption would be related to maximal growth rate [18].

**Fig. 3:**
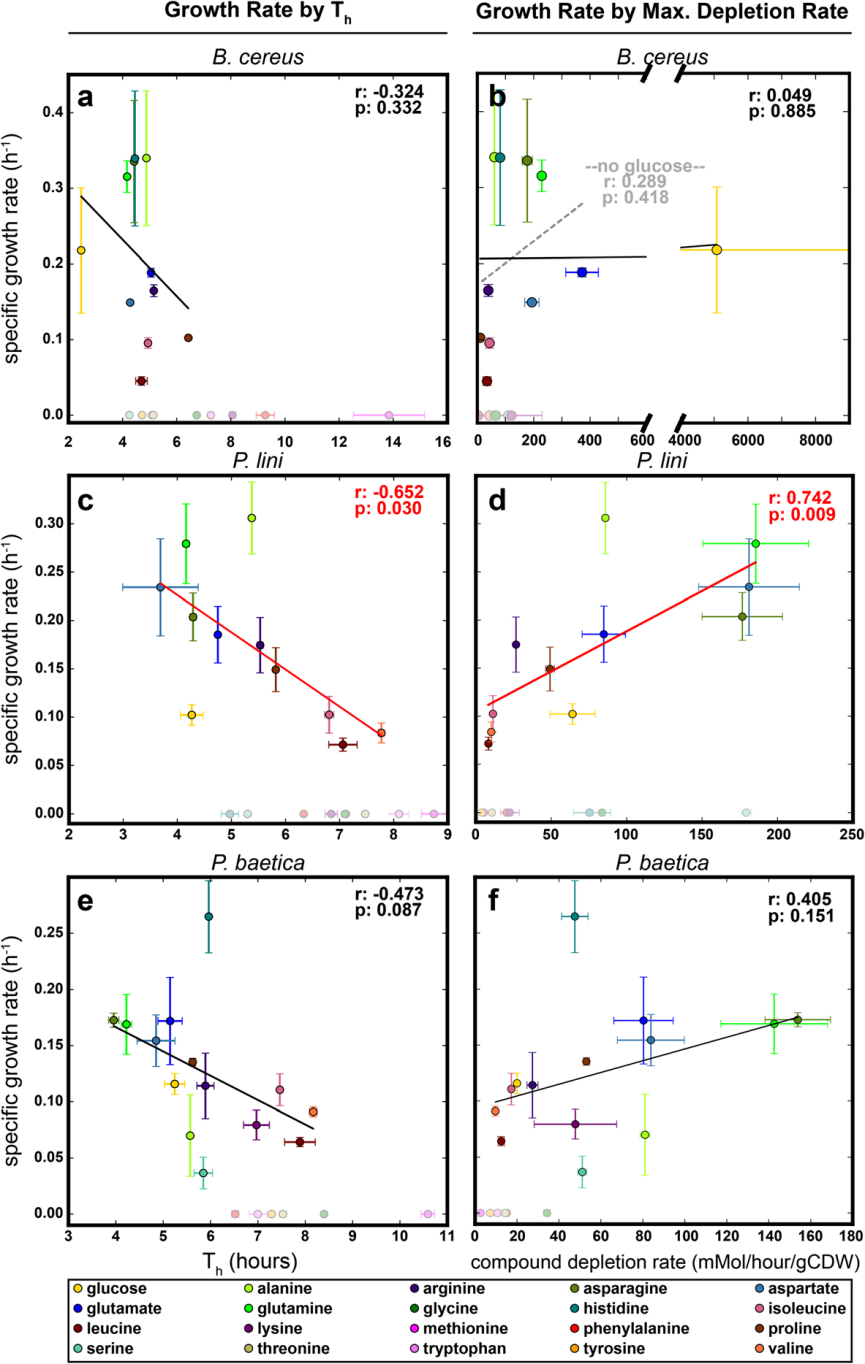
Physiological Correlations. Correlations between specific growth rate on a compound as a sole carbon source, and T_h_ (**a**, **c**, **e**) or maximum compound depletion rate relative to biomass (grams cell dry weight (gCDW)) (**b**, **d**, **f**) in complete defined medium for species *Bc* (**a**, **b**), *Pl* (**c**, **d**) and *Pb* (**e**, **f**). Compounds that did not support growth as a sole carbon source (specific growth rate of zero) are *shaded lighter* at the bottom of each plot. Pearson correlation coefficients (*r*) and *p*-values (*p*) for the set of compounds for which the specific growth rate was nonzero are depicted in the upper-right of each plot. Correlations that had a *p*-value less than 0.05 were *colored red. Error bars* depict standard error

We tested some of these general assumptions by comparing the calculated T_h_ values and maximum usage rate of each compound to the specific growth rate, starting molarity of the compound, and predicted total protein composition of each species, in order to determine what the substrate preference order might be correlated with (Fig. 3 and Additional file 1: Figure S3). The specific growth rate of a species on a compound was determined by growing the species on that compound as a sole carbon source (see Methods). Due to the excess nitrogen added to the medium (see Methods), we do not expect the C:N ratio of a given carbon source to have a significant impact on the growth rate of the organism. Surprisingly, the only significant (*p* < 0.05) correlations between all of these tests were that the specific growth rate of *Pl* on a given compound was weakly correlated with the T_h_ of that compound (*r* = −0.652, *p* = 0.030) and with the maximum depletion rate of that compound relative to biomass (*r* = 0.656, *p* = 0.028) (Fig. 3c and d). These correlations support the common assumptions listed above, especially those rationalizing catabolite repression, as the compound that provides the higher rate of growth is depleted earlier and more rapidly than others. It is interesting that glucose did not confer the fastest specific growth rate for any of the strains, despite glucose generally being considered a superior source of energy. This is not surprising, however, as it is known that pseudomonads preferentially use amino acids over glucose [21]. The rationalization of this phenotype is that in the soil environments where many pseudomonads (and *B. cereus*) live, decomposition products such as amino acids and organic acids are more readily available than sugars [21]. However, the lack of any strong or significant correlations in the bacillus and the other pseudomonad indicates that there are other factors at play that determine an organism’s preferred substrate usage. It is apparent that not all microbes prefer to use substrates sequentially at all; the grouping of substrate utilization by *Bc* is a striking example of this. The resources within the second utilization group (Fig. 2a) conferred a wide range of specific growth rates, from zero to the highest observed for all substrates, and all were utilized within 2 h of each other (Fig. 3a). It is likely the case that the simultaneous usage of these substrates confers the greatest physiological advantage. *Bc* could possess a metabolic strategy that does not perfectly follow the well-established paradigm of catabolite repression. Ultimately, it is clear that bacteria dramatically differ in regulation of catabolite uptake, and it is not prudent to make general assumptions on microbial metabolism based solely on observations from a few model organisms and/or the energetic potential of substrates.

Our experiments to test these correlations yielded a number of interesting results in addition to those described above. First, all three species grew on glucose as the sole carbon source without added amino acids, which was not predicted based on genomic functional predictions. The genomes of these organisms were available in the Integrated Microbial Genomes (IMG) database (img.jgi.doe.gov), where functional predictions are made by associating annotated genes with KEGG Orthology terms and KEGG pathways and MetaCyc reactions [22]. These analyses indicated auxotrophy for lysine, phenylalanine, tyrosine, histidine and serine in the case of *Bc*, and for lysine, histidine, leucine and coenzyme A for *Pl* and *Pb*, meaning these organisms could not grow with a single carbon source, as they would be unable to synthesize those amino acids or cofactors. This observation highlights that all computational predictions should be treated as only suggestions, and should always be tested experimentally before making any assertions. Additionally, there were a number of compounds that did not support growth as sole carbon sources, but were depleted throughout the growth of the species in our complete defined medium (Fig. 3, lightly shaded compounds). This finding indicates that caution should be employed when making physiological assertions based on single-substrate studies, for example those that have individual substrates arrayed in multi-well plates; many microbes can only utilize certain compounds when other substrates are present, the phenomenon of co-metabolism [23]. We should note, however, that we do not know the details of how these compounds are depleted in the rich defined medium, only that they are depleted from the medium; they may simply be exogenously transformed. Finally, we determined the maximum depletion rate of all the substrates by the three species and normalized to grams cell dry weight (gCDW). We observed these rates to be less than 400 mMol/hour/except for glucose depletion by *Bc*, which we calculated to be about 5079 mMol/hour/gCDW (Additional file 3: Table S1). In comparison, various studies of different organisms have measured the glucose uptake rate to range from 2 to 60 mMol/hour/gCDW [24–26]. The depletion of glucose corresponds to rapid loss of signal representing glucose roughly 2.4 h into the growth curve (see Additional file 2), when hardly any biomass has been made. This is likely an artifact of our targeted analysis, as we are not directly observing what is happening to the glucose; extracellular enzymes may convert it to another molecule that is then imported into the cell at a different rate as opposed to the cell transporting glucose directly. This raises the question of why *Bc* would expend extra energy to synthesize these enzymes, when it presumably can use glucose as is. Perhaps it converts glucose to a molecule that is not usable by other species, thus sequestering a valuable energy source and gaining a competitive advantage.

### Predicting consortium metabolism based on models of individual isolates

**Fig. 4:**
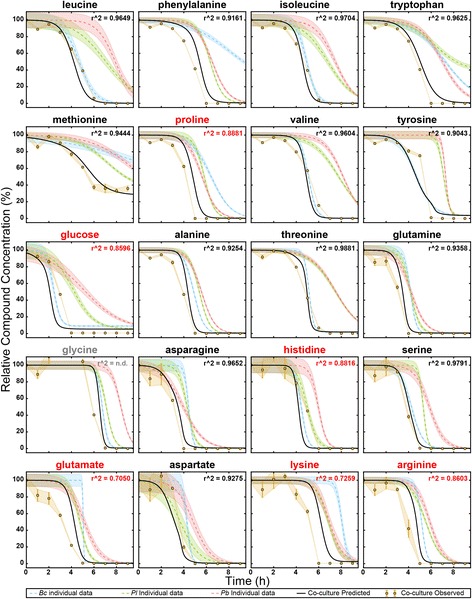
Co-culture observations compared to predictions, normalized to t_0_ concentration of each metabolite. *Blue*, *green* and *red dashed lines* represent the observed depletion of each compound by *Bc*, *Pl* and *Pb*, respectively, when grown in isolation. The *solid black line* is the predicted depletion of a co-culture of all three strains. The *golden circles* represent the measured compound concentration in the co-culture medium. *Error bars* and/or *shading* represent standard error (*n* = 3). Glycine at time point 4 could not be calculated because the measurement was outside the dynamic range of the calibration curve, and the *r* ^*2*^ was not determined (n.d.) for glycine. Non-normalized figure is shown as Additional file 1: Figure S5

Having modeled the substrate usage of each species for each compound, we hypothesized that these models could be combined to predict how a consortium composed of the three species might utilize the substrates. We simulated the time-dependent depletion of each compound by a consortium composed of the bacillus and two pseudomonads (see Methods, Eq. 2 and Algorithm 2). Briefly, the functions describing the compound usage by each species were summed (Additional file 1: Figure S2A), and the time at which this summed use curve reached the total available compound was determined. This time of depletion was then used to predict how much of a given metabolite each species would have utilized when grown in co-culture, and the compound usage by each species was re-modeled (Additional file 1: Figure S2B colored dashed lines) and added together to form the co-culture prediction (Additional file 1: Figure S2B solid black line). These predictive models allowed us to make several hypotheses that are relatively simple to test. First is the usage curve of each metabolite by the co-culture. Related to this, we can predict the time at which all of a given metabolite will be depleted, and when all metabolites will be depleted. From this we predict that 14 compounds will be nearly depleted (less than 10% of starting concentration) by 6 h, and all but methionine will be completely consumed by 9 h (Fig. 4). Based on this, one could reasonably argue that a consortium composed of these three species would reach stationary phase sometime between 6 and 9 h, in contrast to the individual species, which all reached stationary phase after 9 h.

To test our predictions, we inoculated a 3-member co-culture at equal optical density in the defined medium (see Methods), collected supernatant time points every hour, and measured the concentrations of all 20 substrates as described for monocultures. We found that many of our predictions were valid: nearly all compounds (17) were depleted to below 10% of starting concentration by 6 h (Fig. 4, gold) and the co-culture accordingly reached stationary phase at this time as well (Additional file 1: Figure S4), presumably because all available substrates were consumed.

### Compounds that follow the model are evenly shared

When analyzing the kinetics of depletion of the compounds, we observed that many (13) compounds agreed very well with the prediction, having *R* ^*2*^ values greater than 0.9 (Fig. 4). Most of the compounds with high *R* ^*2*^ values began to decrease slightly earlier or at a slightly faster rate than predicted, which could be attributed to experimental error in initial culture density. However, the depletion of most compounds were still very close to the predicted model, indicating that the shared usage between the species could be very close to “blind” conditions, where the presence of other species does not affect the substrate usage decisions of each individual species. It is important to note that the high substrate concentrations likely explain the successful predictions using this simple modeling approach. Specifically, the substrate concentrations, initially at high micromolar concentrations, are likely well above the K_m_ for the transporters and rate-limiting enzymes. For example many bacterial amino acid transporters have K_m_ values in the low micromolar range [27, 28], such that the transporters and enzymes are saturated. We anticipate that much more detailed models accounting for substrate concentration would be required at soil- and groundwater-relevant substrate concentrations, which can be as low as 0.5–10% of the concentrations used in this study ([29] and Jenkins et al., in preparation).

### Compounds that deviate from the model

The remaining 7 compounds (glucose, histidine, glutamate, lysine, arginine, proline and glycine) deviated significantly from our predictions (*R* ^*2*^ < 0.9) (Fig. 4, red text), suggesting some sort of interspecies interaction (s) is/are present that affect the depletion of those compounds. These interactions could include both direct interactions (e.g. signaling molecules) or indirect (e.g. the effect one species has on the medium). Among indirect interactions, metabolites secreted by one species that were not measured in this study (e.g. overflow metabolites such as acetate) could be consumed by another species, thus altering its resource usage, or could inhibit a certain metabolic pathway or even enhance the degradation of a metabolite due to co-metabolism.

Glucose and histidine were both depleted more slowly than predicted. The simplest explanations for this are that the metabolic systems that deplete these compounds are indeed concentration dependent, or that these compounds are secreted by at least one member in the co-culture, resulting in an apparent slowdown of net depletion. Another possibility for this would be that there is a buildup of product in the co-culture that exerts feedback inhibition on the metabolism of these two compounds.

In contrast, glycine, proline, lysine, arginine and glutamate were all depleted faster than predicted. This is more difficult to explain and suggests at least one microbe has altered its phenotype due to the presence of other microbes, or that other exometabolites are influencing consortial behavior. For example, one species may have up-regulated metabolic pathways involving these compounds in an effort to outcompete others, either for the purpose of direct competition for the substrate, or in order to synthesize antibiotic compounds [30]. Alternatively, co-culturing of these microbes has resulted in an emergent function of increased flux of the substrate (s) through the system. This could be due to a cross-feeding effect where one microbe depletes an inhibitory compound of another microbe or one microbe’s products induce the co-metabolism of that product and one of these substrates. Testing these hypotheses would require an extensive untargeted metabolomics study, an extremely interesting direction for future studies.

## Conclusions

This study examining substrate competition for 20 abundant substrates by 3 species demonstrates that at least some portion of the metabolic behavior of a microbial consortium can be predicted by measuring the metabolism of microbes grown in monoculture. This likely can also apply to more complex situations, for example separately measuring the metabolism of an existing microbial community and a foreign isolate, and predicting what the metabolic function might be if the isolate were introduced into the community. In any system, compounds that do not fit the predictions indicate emergent functions of the coculture and may highlight substrates that are somehow affected by species-species interactions. These may be occurring passively in the cases of feedback inhibition and co-metabolism, or actively in the case of one species altering its phenotype in order to outcompete others. Further studying these outlier substrates can shed light on metabolic interactions between microbes within a community. Finally, by studying the growth kinetics on varying levels of substrates, growth models based on the Monod equation can be generated and used to predict relative species abundance in these co-cultures. Ultimately, incorporating this predictive strategy when studying community metabolisms can help pinpoint interesting biological questions, as well as aid in the design of synthetic consortia.

## Methods

### Isolates and identification

The 16S rRNA gene for each isolate was amplified using primers 27 F (AGAGTTTGATCMTGGCTCAG) and 1492R (CGGTTACCTTGTTACGACTT), and sequenced at the Eurofins sequencing facility (Eurofins MWG Operon LLC, Louisville, KY). Forward and reverse sequences were manually merged and used as queries using nucleotide BLAST against the 16S rRNA sequence database at NCBI.

### Phylogenetic tree construction

16S rRNA gene sequences were obtained from IMG (img.jgi.doe.gov), except for *B. cereus*, *P. lini* and *P. baetica*, which were directly sequenced (see above). Gene sequences were aligned using MUSCLE [31, 32], curated using GBlocks [33], and the tree was constructed using PhyML [34] with 100 bootstraps, using the phylogeny.fr web server [35, 36]. The final tree was rendered using FigTree (http://tree.bio.ed.ac.uk/software/figtree/).

### Growth medium and culturing

All bacterial species were initially inoculated from frozen glycerol stocks onto an R2A agar plate prepared using Difco R2A Agar (BD, Franklin Lakes, NJ) and incubated overnight at 30 °C. The medium used for metabolomics experiments consisted of 1× Wolfe’s vitamins and 1× Wolfe’s minerals solutions [37], 1.5 mg/mL ammonium chloride, 0.6 mg/mL potassium phosphate, and 0.1 mg/mL each of D-glucose and the following L-amino acids: alanine, aspartate, glutamate, phenylalanine, glycine, histidine, isoleucine, lysine, leucine, methionine, asparagine, proline, glutamine, arginine, serine, valine, threonine and tryptophan. Tyrosine was additionally supplied at 0.01 mg/mL. Species were individually cultured in 5 mL of this medium overnight at 30 °C from the R2A plate, then washed 3× by centrifugation at 5000 xg and resuspending in fresh medium. Washed cells were used to inoculate 50 mL of the medium in 250 mL Erlenmeyer flasks, at an initial optical density (OD_600_) of 0.012–0.017 as measured by a SpectraMax Plus 384 plate reader. These cultures were incubated at 30 °C, shaking at 200 rpm. Biomass was monitored by OD_600_ measurements, with grams cell dry weight (gCDW) inferred by a predetermined correlation factor, *k* with units gCDW/L/OD. To determine *k*, gCDW was measured from four cell suspensions of each species that were harvested by centrifugation and washed in phosphate buffered saline solution (Sigma-Aldrich). An example calculation is as follows: 1 mL of OD_600_ 0.496 corresponded to 0.0005 gCDW. 0.0005 gCDW/.001 L/0.496 OD_600_ = 0.9 gCDW/L/OD. gCDW for each time point was calculated by multiplying *k* by the measured OD_600_ and the volume of the culture (50 mL subtracted by the volume of culture removed for sampling).

For co-culture experiments, 50 mL cultures were inoculated with an OD_600_ of 0.012 of each species, resulting in an initial co-culture density of 0.036. 200 μL of cell culture was aspirated for OD_600_ measurements taken in a 96-well Falcon tissue culture plate with flat bottom. For all growth experiments, the water used to prepare the medium and uninoculated medium were incubated alongside the experimental flasks, as controls.

Growth assays of species on individual carbon sources were performed in 96-well Falcon tissue culture plates with flat bottom and low evaporation lid, in a total volume of 200 μL. The medium consisted of the same concentrations of Wolfe’s vitamins and minerals, ammonium chloride and potassium phosphate. Individual carbon sources were added at a concentration of 0.5 mg/mL. Species were pre-cultured and washed as before, and wells were inoculated at an OD_600_ of 0.05. The plates were incubated at 30 °C, shaking at “medium” speed in BioTek Synergy HT and Tecan Infinite F200 Pro plate readers, for 48 h.

### Metabolomics sample extraction

Hourly time points of 1 mL of cell culture and controls (see above) were aspirated and centrifuged at 5000 xg to pellet the cells. 800 μL was aspirated from the top, taking care not to disturb the cell pellet, and split into two 400 μL aliquots, which were immediately frozen at −80 °C. A calibration curve was created with the medium used for culturing: 1× culture medium, 1/2×, 1/10×, 1/100×, 1/1000× and 1/10000× dilutions were prepared using culture medium without any carbon sources as the diluent. All experimental, control and calibration curve samples were lyophilized overnight, and metabolites were extracted in 300 μL methanol with 25 μM ^13^C-phenylalanine for use as an internal standard. Final extracted samples were stored in Agilent 96-well sample plates and immediately analyzed via LCMS or stored at −80 °C.

### Metabolomics data acquisition and quantification

An Agilent 1290 LC system equipped with a ZIC-pHILIC column (150 mm × 2.1 mm, 5 μm 100 Å, Merck SeQuant) was used for metabolite separation with the following LC conditions: solvent A, 5 mM ammonium acetate; solvent B, 9:1 acetonitrile:H_2_O with 5 mM ammonium acetate; flowrate: 0.25 mL/min; timetable: 0 min at 100% B, 1.5 min at 100% B, 25 min at 50% B, 26 min at 35% B, 32 min at 35% B, 33 min at 100% B, and 40 min at 100% B; column compartment temperature of 40 °C. Mass spectrometry analyses were performed using Agilent 6460 triple quadrupole mass spectrometer. Agilent software (Santa Clara, CA): Optimizer was used for establishing fragmentor and collision cell voltages as well as precursor and product ion transitions while Mass Hunter QQQ Quantitative Analysis (version 6.0) was used for compound quantification. Retention times, collision energies and transitions for each compound are listed in Additional file 1: Table S2.

### Substrate depletion modeling

The Anaconda package and custom IPython notebooks were used for all computational tasks [38], which are publicly available at https://github.com/biorack in the “Predicting metabolic properties of a microbial co-culture” repository. Data were stored and organized using Pandas [39] and NumPy [40], and graphs created using Matplotlib [41]. Metabolite depletion was modeled using leastsq from scipy.optimize [42], fitting the data to the Behrends model (Eq. 1):

1 $$ \xi =\frac{a}{1+{e}^{\frac{t-{t}_{50}}{w}}}+o $$

Where *a* is amplitude and *o* is offset (see Fig. 1). These two parameters were defined from the data: amplitude was defined to be the average of the *t* = 0 data point and the maximum value data point in the data set of each compound, and offset was defined as the lowest value in the data set. All other parameters were solved using leastsq, with the criteria that they had to be positive values. The exact steps are shown in Algorithm 1:

T_h_ and usage window values were calculated from the Behrends model. All correlation coefficients and *p*-values were calculated using the pearsonr function in the stats package of scipy.

### Co-culture predictions

The equations representing the depletion of a compound (depletion curve) by a species were subtracted from the initial starting concentration of the compound, creating an expression that represented the amount of compound used by each species over time (usage curve); these are the curves shown in Additional file 1: Figure S2A. These expressions were summed to generate an approximate total usage curve, and the time at which this curve crossed the total amount of available compound was determined: the time when all available compound has been used $\left({t}_{d_j}\right)$. The amount of available compound was defined to be the starting concentration of a compound minus the lowest offset parameter between the three species, as the species with the lowest offset parameter for a substrate will presumably deplete the substrate to that level, but not more, even in a co-culture. The ${t}_{d_j}$ was used to approximate the amount of compound that each species would have consumed by that time. The individual usage curves were capped at this compound level at this time and subtracted from the starting concentration of compound to revert the curves back to depletion curves. These new curves were used to solve new parameters for the Eq. 1, generating new models of compound depletion in mixed conditions. These new models were then summed, producing the predicted total co-culture usage of each compound. This can be summarized by the general Eq. 2:

2 $$ C-{\displaystyle \sum_i^{species}}C-\frac{a_{ij}\hbox{'}}{1+{e}^{\frac{t-{t}_{50_{ij}}\hbox{'}}{w_{ij}\hbox{'}}}}+{o}_{ij}\hbox{'} $$

where C is the total amount of substrate *j* that is available to the mixed culture of set *species*. This is defined as the starting concentration of *j* minus the smallest *o*_*j*_ in *species.a*_*ij*_ ', *o*_*ij*_^′^, ${t}_{5{ 0}_{ij}}$, and *w*_*ij*_ ', are parameters that describe the depletion of *j* by species *i* in the co-culture of the individual in the set *species*, shown in Algorithm 2:

## Additional files

Additional file 1: Supplemental Table 2 and Supplemental Figures 1-5. (PDF 781 kb)
Additional file 2: Raw data and fitted curves for levels of each metabolite during isolate growth curves. (PDF 2162 kb)
Additional file 3: Supplemental Table 1. (XLSX 55 kb)

## Abbreviations

Bc: *Bacillus cereus*
gCDW: Grams cell dry weight
IMG: Integrated microbial genomics website (img.jgi.doe.gov)
K_m_: Michaelis constant
LC-MS: Liquid chromatography coupled with mass spectrometry
OD_600_: Optical density at 600 nm
Pb: *Pseudomonas baetica*
Pl: *Pseudomonas lini*
T_h_: Time of half depletion of a resource
