# Supplementary material for: Dynamic substrate preferences predict metabolic properties of a simple microbial consortium
Source: BMC Bioinformatics. 2017 Jan 23;18:57. doi: 10.1186/s12859-017-1478-2 (PMC5259839; doi:10.1186/s12859-017-1478-2)
Supplement: Additional file 1: — Supplemental Table 2 and Supplemental Figures 1-5. (PDF 781 kb) [file 12859_2017_1478_MOESM1_ESM.pdf]

Table S2. Dynamic Multiple Reaction Monitoring (dMRM) data acquisition table.

Compound Name	Precursor Ion	Product Ion	Ret Time (min)	Fragmentor	Collision Energy	Cell Accelerator Voltage	Polarity	ion type
alanine	90.1	45.2	11.523	45	39	4	Positive	quantifier
alanine	90.1	44.3	11.523	45	19	4	Positive	qualifier
arginine	175.1	70.2	23.36	99	23	4	Positive	quantifier
arginine	175.1	60.2	23.36	99	19	4	Positive	qualifier
asparagine	133.1	74.2	13.292	89	19	4	Positive	quantifier
asparagine	133.1	46.2	13.292	89	19	4	Positive	qualifier
aspartate	134	88.1	14.653	84	15	4	Positive	quantifier
aspartate	134	74.2	14.653	84	15	4	Positive	qualifier
aspartate	134	74.1	14.653	89	19	4	Positive	qualifier
glucose	203.1	203.1	11.082	0	0	4	Positive	quantifier
glucose	203.1	143	11.082	147	15	4	Positive	qualifier
glucose	203.1	101	11.082	147	19	4	Positive	qualifier
glucose	203.1	83	11.082	147	23	4	Positive	qualifier
glutamate	148.1	102	14.087	89	15	4	Positive	quantifier
glutamate	148.1	84.2	14.087	89	19	4	Positive	qualifier
glutamate	148.1	56.2	14.087	89	31	4	Positive	qualifier
glutamine	147.1	130	12.797	94	15	4	Positive	qualifier
glutamine	147.1	84.2	12.797	94	19	4	Positive	quantifier
glycine	76	31	12.77	50	35	4	Positive	quantifier
glycine	76	30.3	12.77	50	15	4	Positive	qualifier
heavy-phe	176.1	129	7.967	94	19	4	Positive	quantifier
heavy-phe	176.1	83	7.967	94	47	4	Positive	qualifier
histidine	156.1	110	13.222	104	15	4	Positive	quantifier
histidine	156.1	83.2	13.222	104	27	4	Positive	qualifier
isoleucine	132.1	86.2	8.361	59	15	4	Positive	quantifier
isoleucine	132.1	69.2	8.361	59	19	4	Positive	qualifier
leucine	132.1	86.2	7.952	70	15	4	Positive	quantifier
leucine	132.1	44.3	7.952	70	23	4	Positive	qualifier
lysine	147.1	84.2	22.928	104	19	4	Positive	quantifier
lysine	147.1	56.2	22.928	104	35	4	Positive	qualifier
methionine	150.1	61.2	9.124	94	27	4	Positive	quantifier
methionine	150.1	56.2	9.124	94	19	4	Positive	qualifier
phenylalanine	166.1	120	7.967	94	19	4	Positive	quantifier
phenylalanine	166.1	77.2	7.967	94	43	4	Positive	qualifier
proline	116.1	70.2	9.234	99	19	4	Positive	quantifier
proline	116.1	43.3	9.234	99	35	4	Positive	qualifier
serine	106	60.2	13.368	55	15	4	Positive	quantifier
serine	106	42.2	13.368	55	23	4	Positive	qualifier
threonine	120.1	74.2	11.992	89	15	4	Positive	quantifier
threonine	120.1	56.2	11.992	89	19	4	Positive	qualifier
tryptophan	103.1	77.2	9.077	191	19	4	Positive	quantifier
tryptophan	103.1	50.2	9.077	191	55	4	Positive	Qualifier
tyrosine	182.1	136	10.595	94	19	4	Positive	Quantifier
tyrosine	182.1	123	10.595	94	15	4	Positive	Qualifier
valine	118.1	72.2	9.454	65	15	4	Positive	quantifier
valine	118.1	55.2	9.454	65	23	4	Positive	qualifier

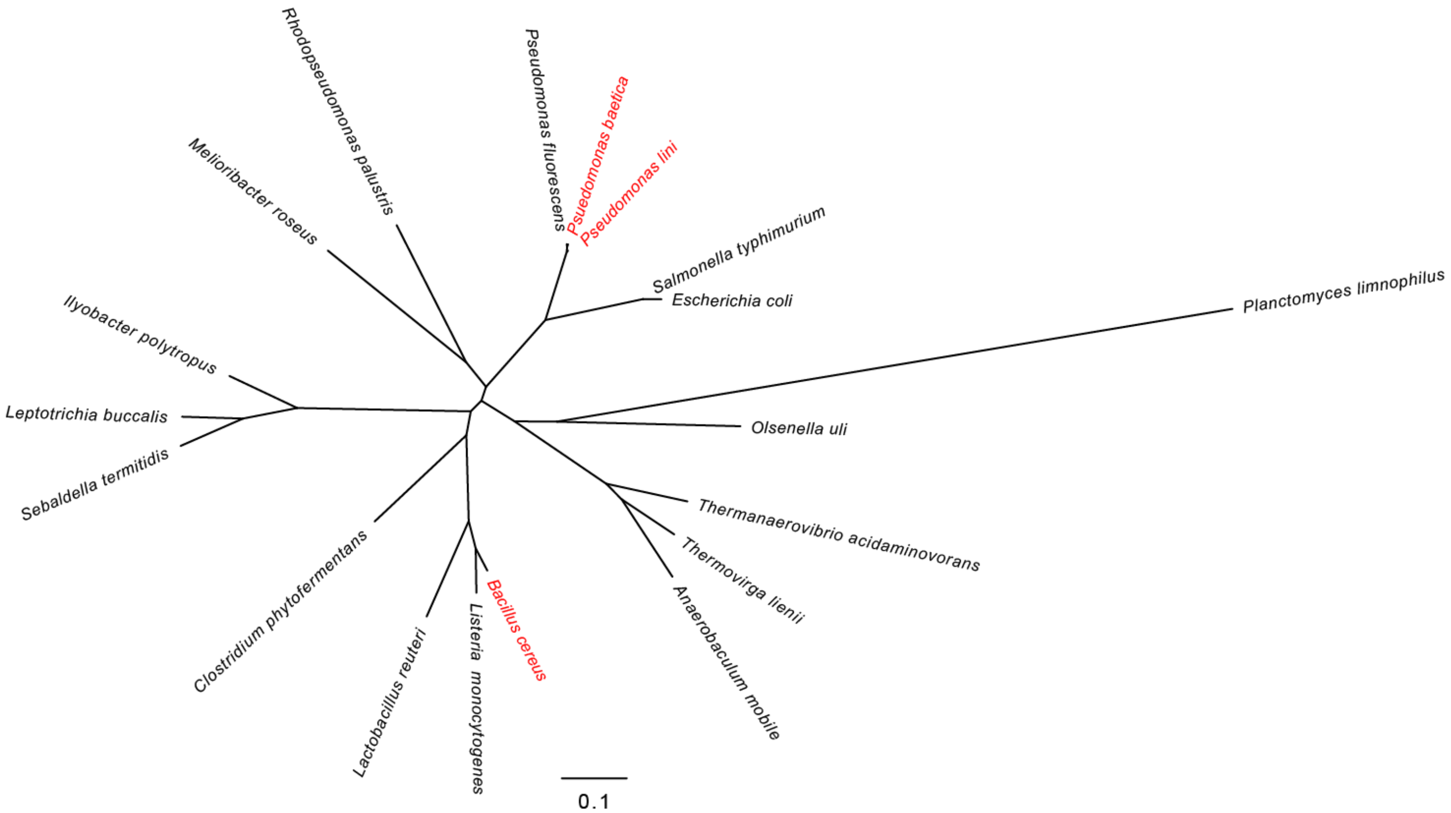

Figure S1. 16S rRNA tree. Species used in this study are highlighted in red.

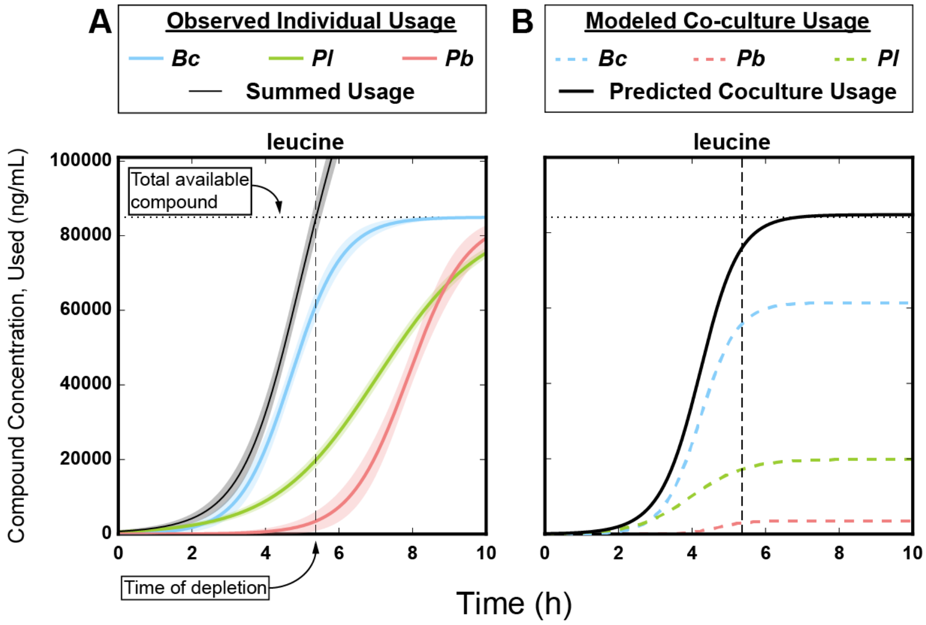

Figure S2. Example prediction development for leucine. A) Colored lines represent the use of the compound over time by each strain. The solid black line is the addition of all three curves. The horizontal dashed line represents the highest amount of compound available to the strains, and the vertical dashed line is the time at which the additive usage reaches this amount, predicting the time of depletion: when all available resource has been consumed. This time was used to approximate the amount of compound that each strain consumed up to that point, which was used to model the dashed lines in **(B)**. **B)** Dashed colored lines represent the predicted usage curve of individual strains when grown in coculture, the sum of which is depicted by the solid black line; the predicted overall usage of the co-culture.

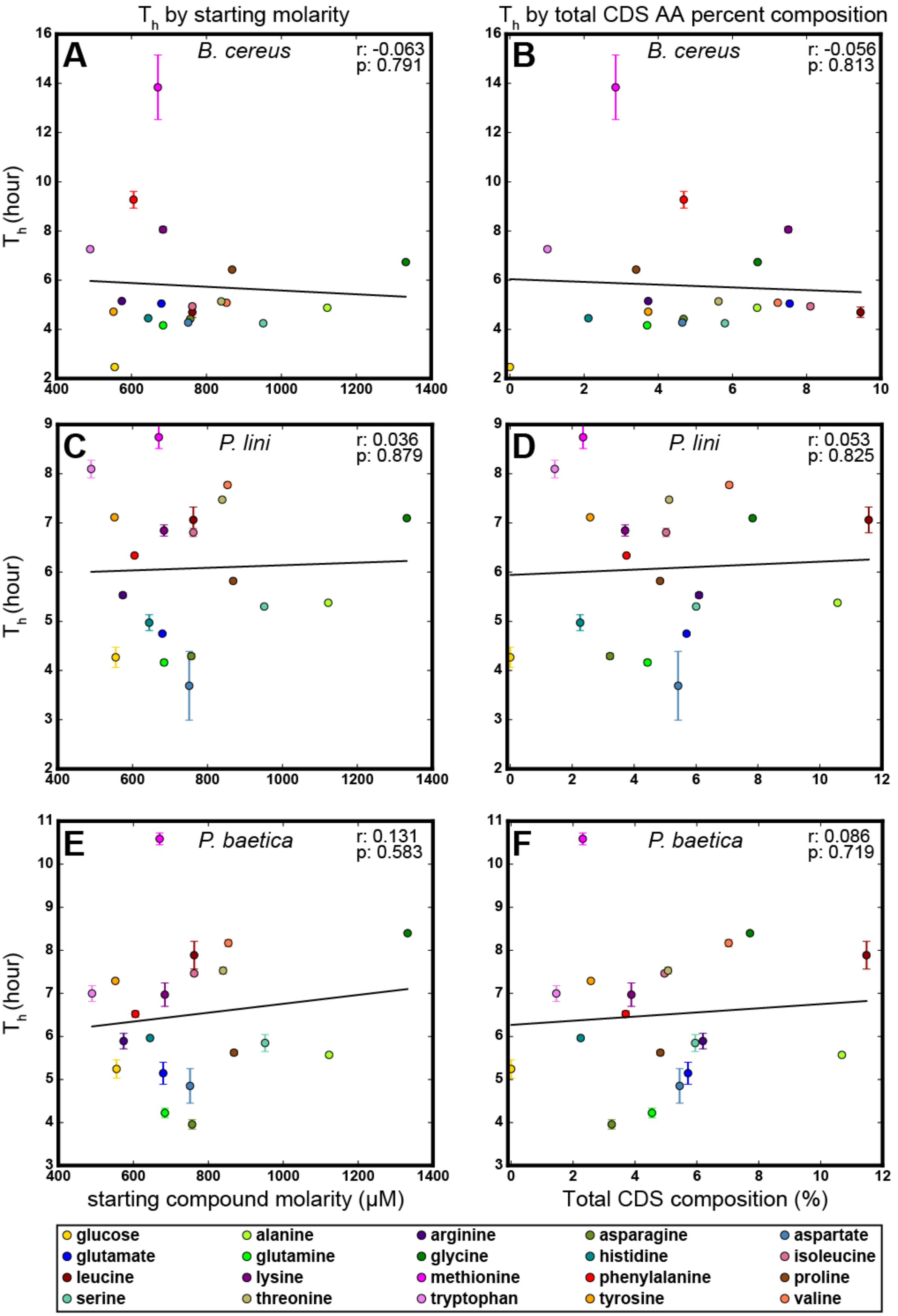

Figure S3. Correlation tests between Th and starting molarity of compounds (A**, C, E) and** percent amino acid composition of all predicted genes in the closes sequenced genome (B, D, F) for *Bc* **(A, B)**, *Pl* **(C, D),** and *Pb* **(E, F).** Pearson correlation coefficients (r) and p-values (p) are shown in the upper right-hand corner of each plot.

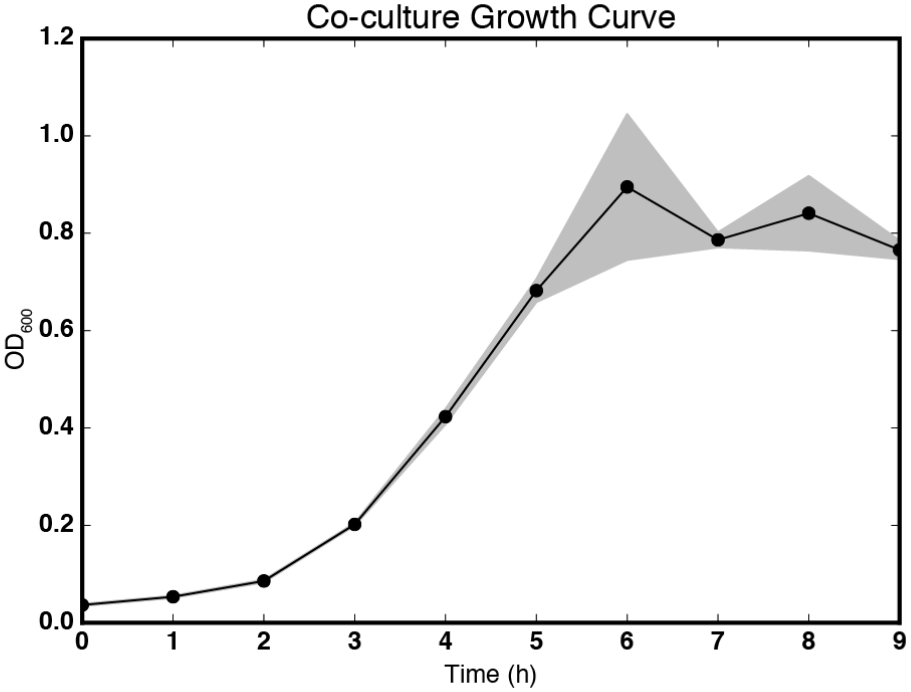

Figure S4. Growth curve of co-culture. Shaded region represents standard deviation (n=3)

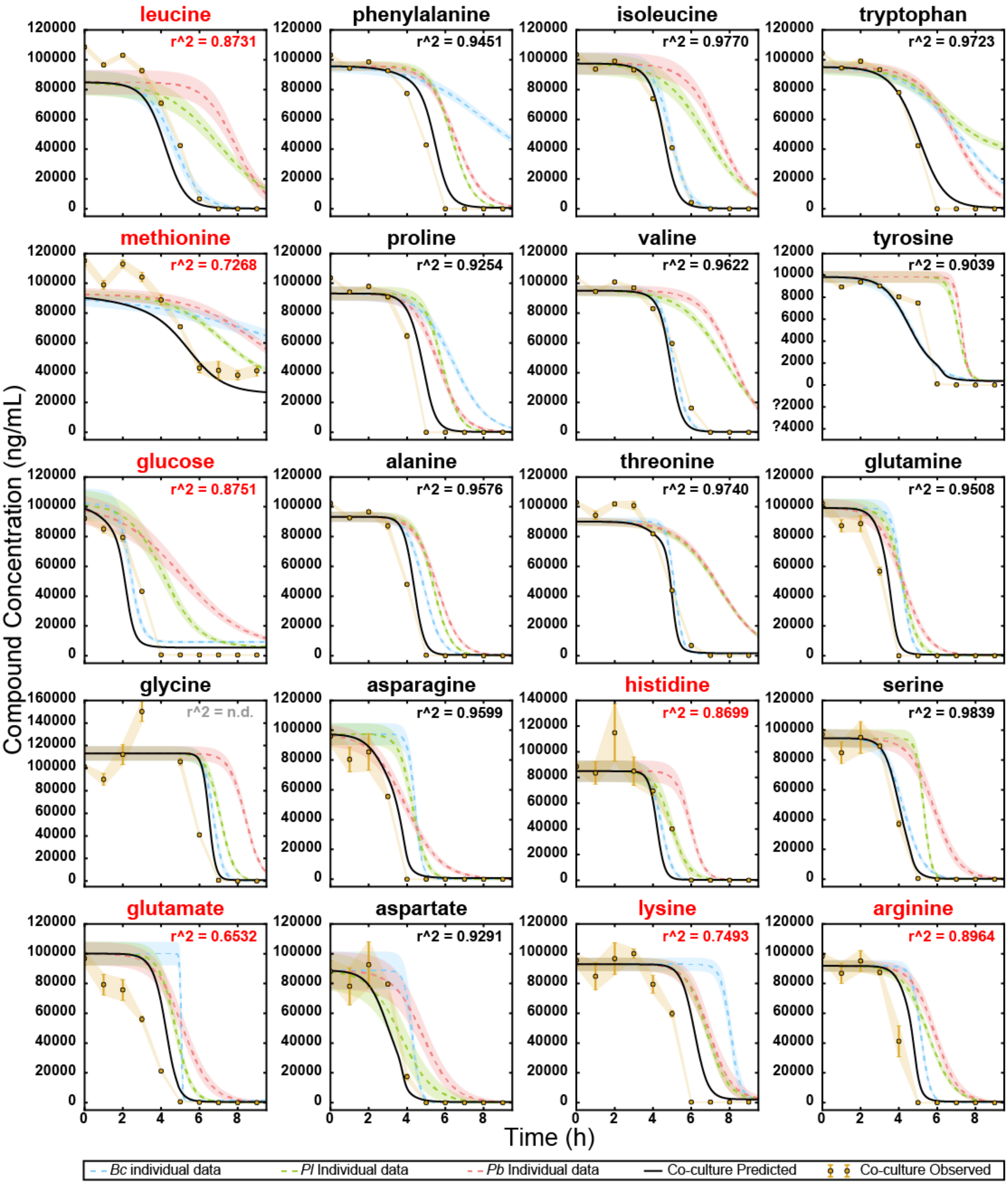

Figure S5. Co-culture observations compared to individuals' data and coculture prediction, not normalized to starting conditions.
