# Supplementary material for: Dynamic substrate preferences predict metabolic properties of a simple microbial consortium
Source: BMC Bioinformatics. 2017 Jan 23;18:57. doi: 10.1186/s12859-017-1478-2 (PMC5259839; doi:10.1186/s12859-017-1478-2)
Supplement: Additional file 2: — Raw data and fitted curves for levels of each metabolite during isolate growth curves. (PDF 2162 kb) [file 12859_2017_1478_MOESM2_ESM.pdf]

Bc, replicate 1

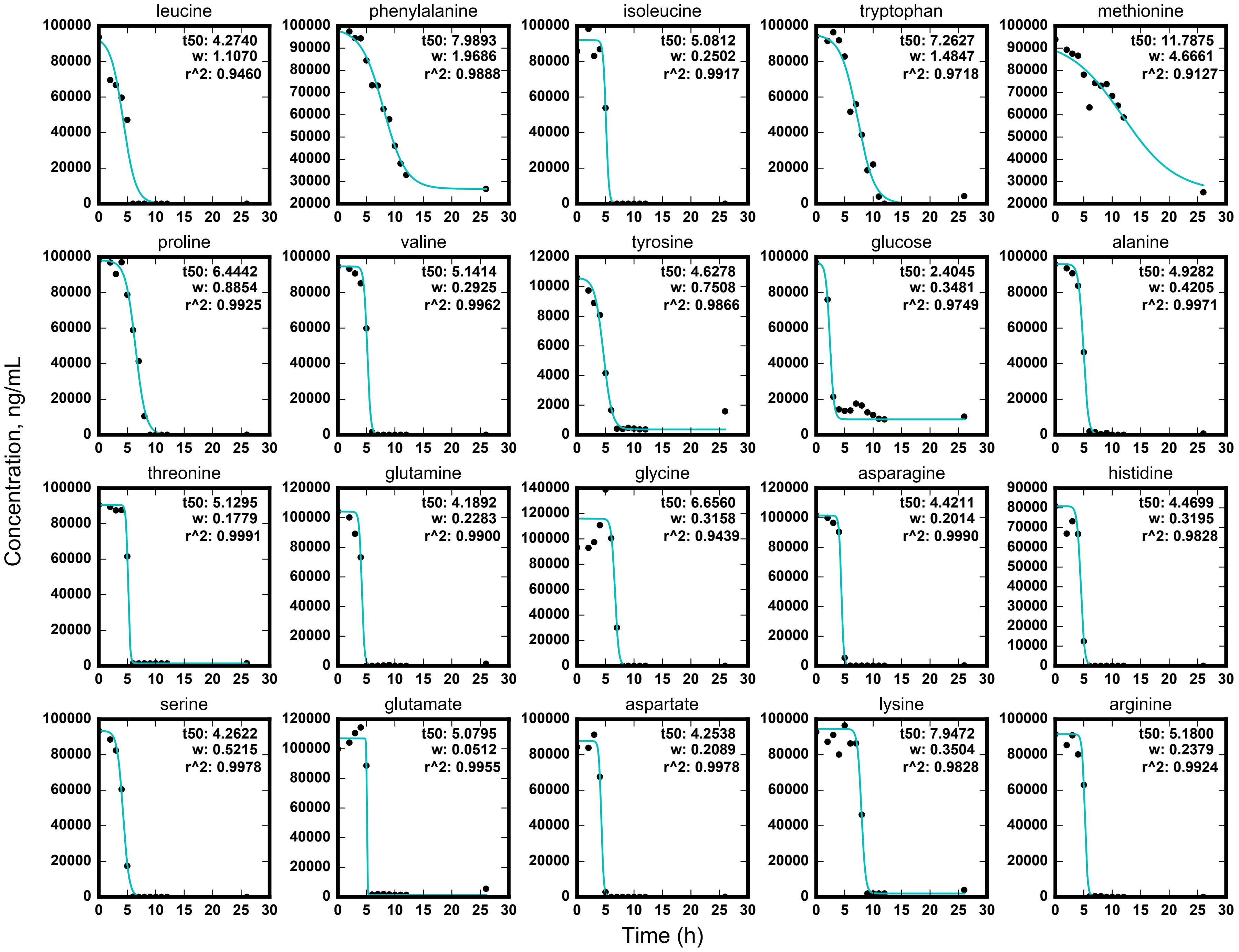

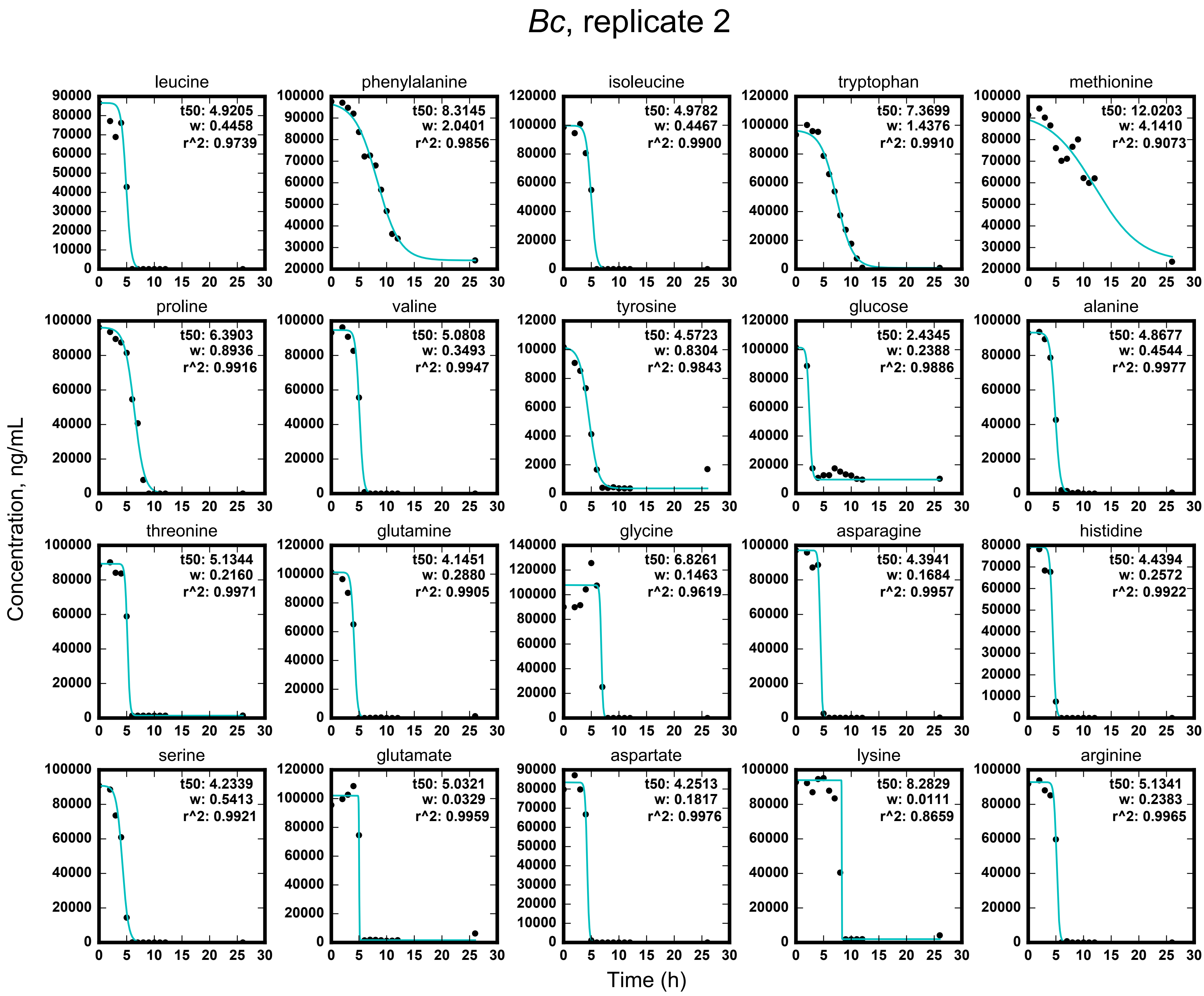
Bc, replicate 2
leucine
t50: 4.9205
w: 0.4458
r^2: 0.9739
phenylalanine
t50: 8.3145
w: 2.0401
r^2: 0.9856
isoleucine
t50: 4.9782
w: 0.4467
r^2: 0.9900
tryptophan
t50: 7.3699
w: 1.4376
r^2: 0.9910
methionine
t50: 12.0203
w: 4.1410
r^2: 0.9073
proline
t50: 6.3903
w: 0.8936
r^2: 0.9916
valine
t50: 5.0808
w: 0.3493
r^2: 0.9947
tyrosine
t50: 4.5723
w: 0.8304
r^2: 0.9843
glucose
t50: 2.4345
w: 0.2388
r^2: 0.9886
alanine
t50: 4.8677
w: 0.4544
r^2: 0.9977
threonine
t50: 5.1344
w: 0.2160
r^2: 0.9971
glutamine
t50: 4.1451
w: 0.2880
r^2: 0.9905
glycine
t50: 6.8261
w: 0.1463
r^2: 0.9619
asparagine
t50: 4.3941
w: 0.1684
r^2: 0.9957
histidine
t50: 4.4394
w: 0.2572
r^2: 0.9922
serine
t50: 4.2339
w: 0.5413
r^2: 0.9921
glutamate
t50: 5.0321
w: 0.0329
r^2: 0.9959
aspartate
t50: 4.2513
w: 0.1817
r^2: 0.9976
lysine
t50: 8.2829
w: 0.0111
r^2: 0.8659
arginine
t50: 5.1341
w: 0.2383
r^2: 0.9965
Concentration, ng/mL
Time (h)

Bc, replicate 3

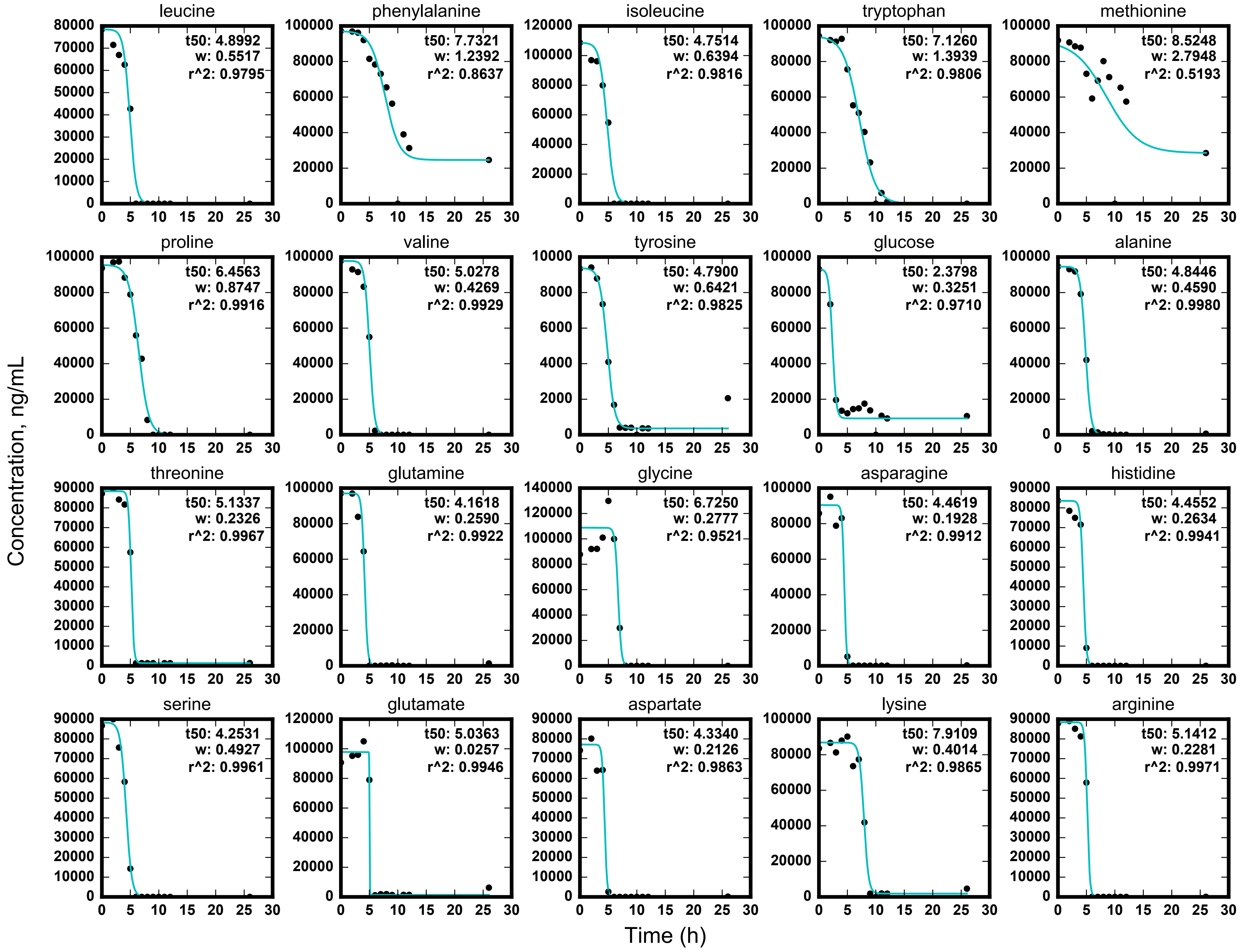

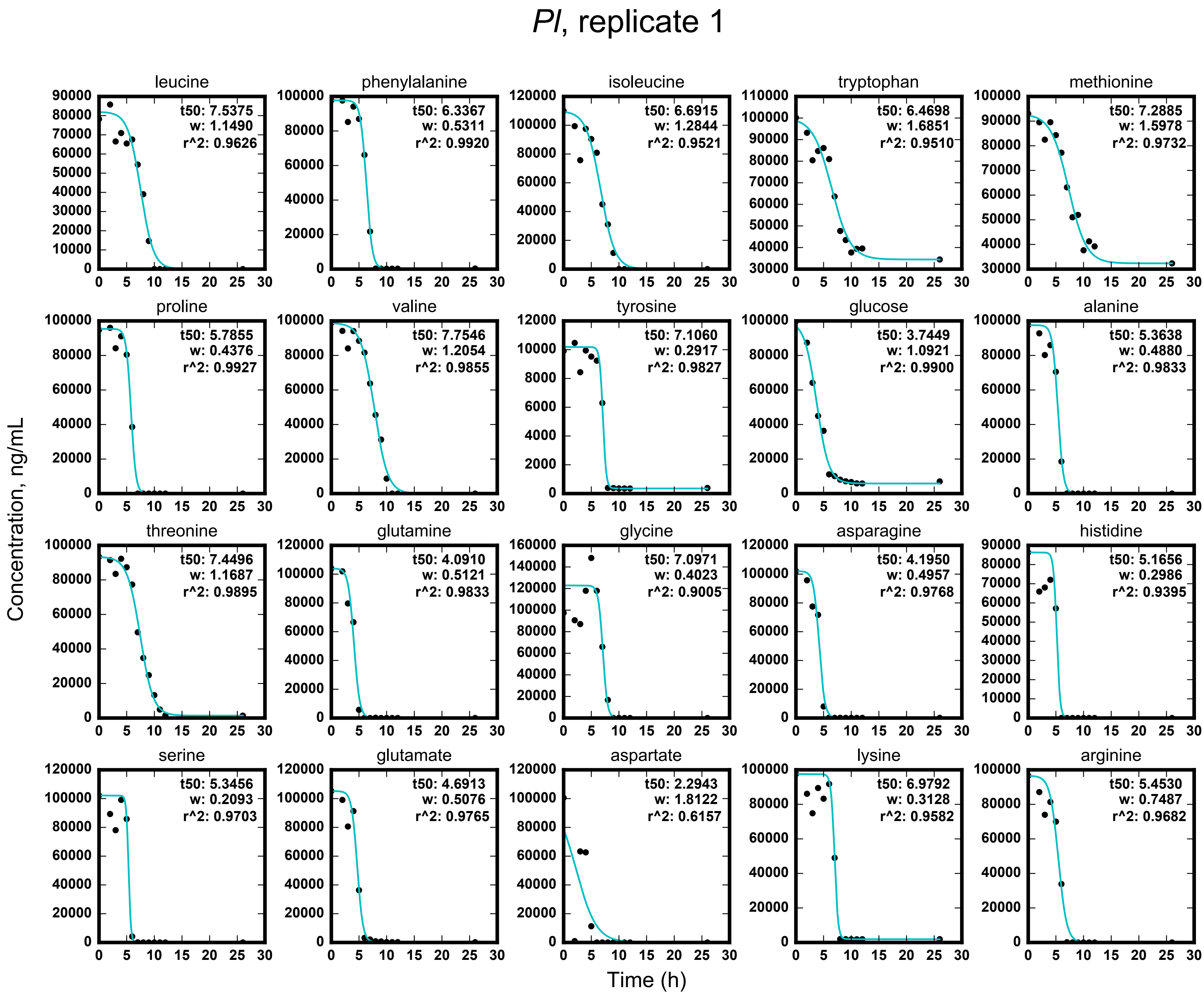

Pl, replicate 1
Concentration, ng/mL
Time (h)
leucine
t50: 7.5375
w: 1.1490
r^2: 0.9626
phenylalanine
t50: 6.3367
w: 0.5311
r^2: 0.9920
isoleucine
t50: 6.6915
w: 1.2844
r^2: 0.9521
tryptophan
t50: 6.4698
w: 1.6851
r^2: 0.9510
methionine
t50: 7.2885
w: 1.5978
r^2: 0.9732
proline
t50: 5.7855
w: 0.4376
r^2: 0.9927
valine
t50: 7.7546
w: 1.2054
r^2: 0.9855
tyrosine
t50: 7.1060
w: 0.2917
r^2: 0.9827
glucose
t50: 3.7449
w: 1.0921
r^2: 0.9900
alanine
t50: 5.3638
w: 0.4880
r^2: 0.9833
threonine
t50: 7.4496
w: 1.1687
r^2: 0.9895
glutamine
t50: 4.0910
w: 0.5121
r^2: 0.9833
glycine
t50: 7.0971
w: 0.4023
r^2: 0.9005
asparagine
t50: 4.1950
w: 0.4957
r^2: 0.9768
histidine
t50: 5.1656
w: 0.2986
r^2: 0.9395
serine
t50: 5.3456
w: 0.2093
r^2: 0.9703
glutamate
t50: 4.6913
w: 0.5076
r^2: 0.9765
aspartate
t50: 2.2943
w: 1.8122
r^2: 0.6157
lysine
t50: 6.9792
w: 0.3128
r^2: 0.9582
arginine
t50: 5.4530
w: 0.7487
r^2: 0.9682

Pl, replicate 2

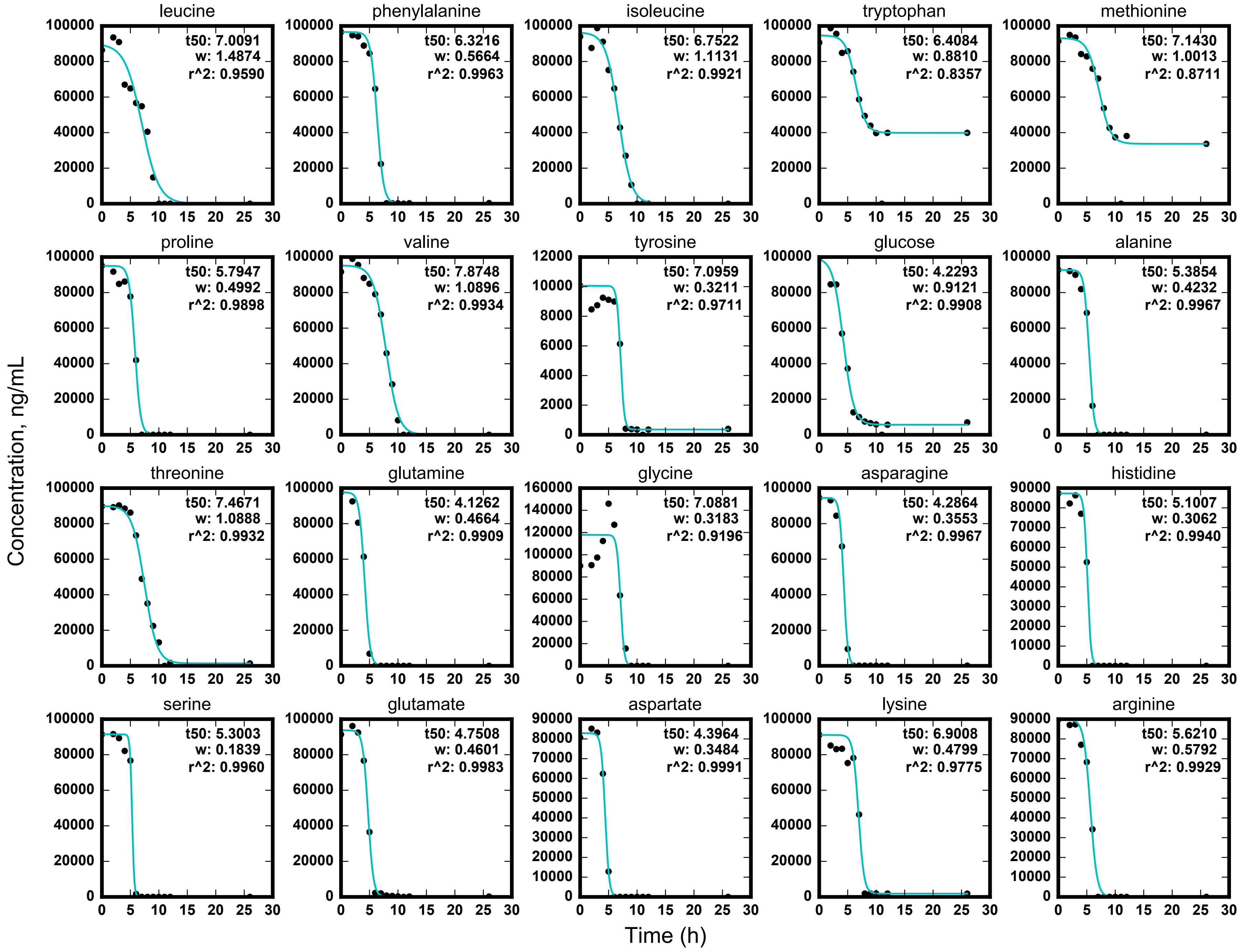

Pl, replicate 3

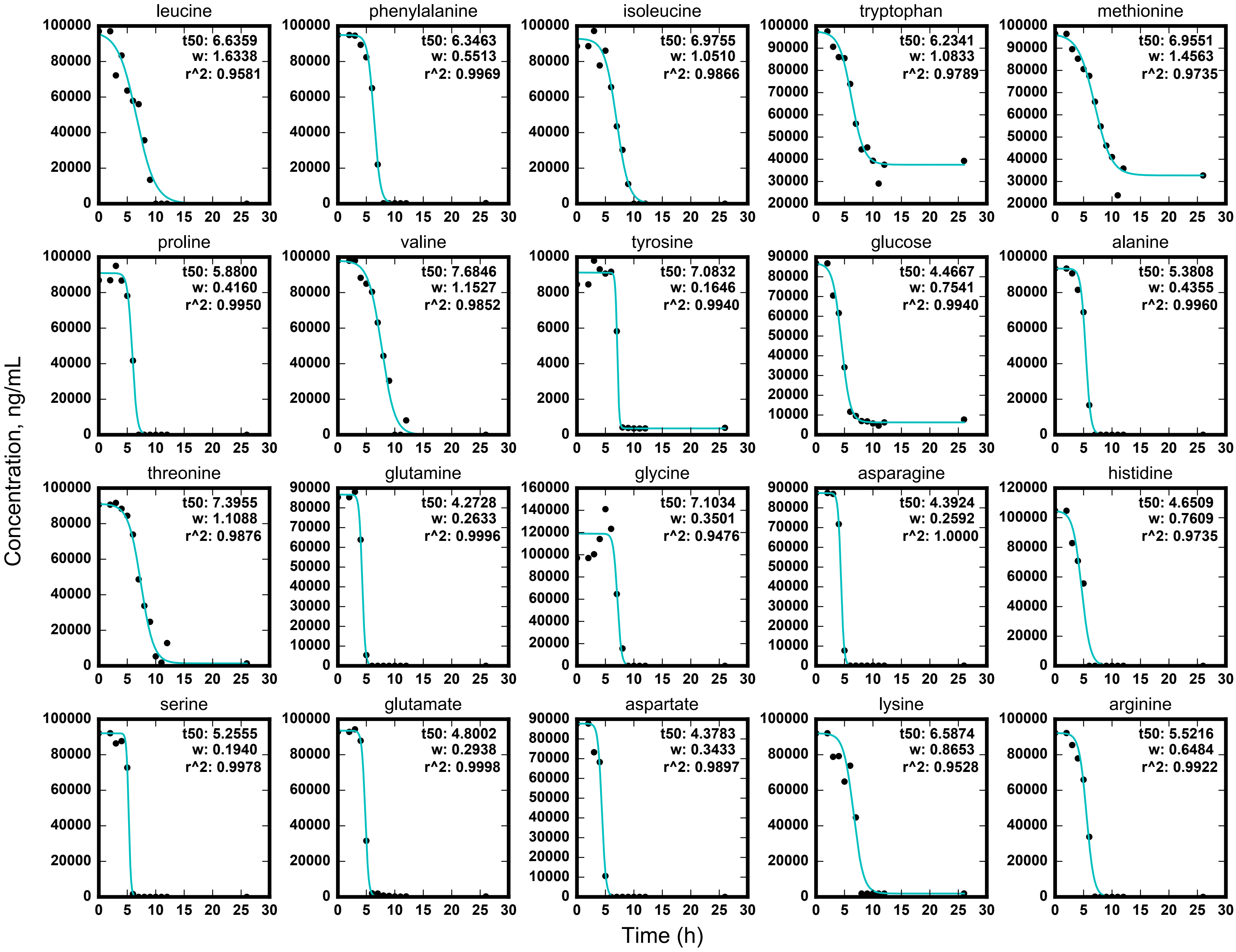

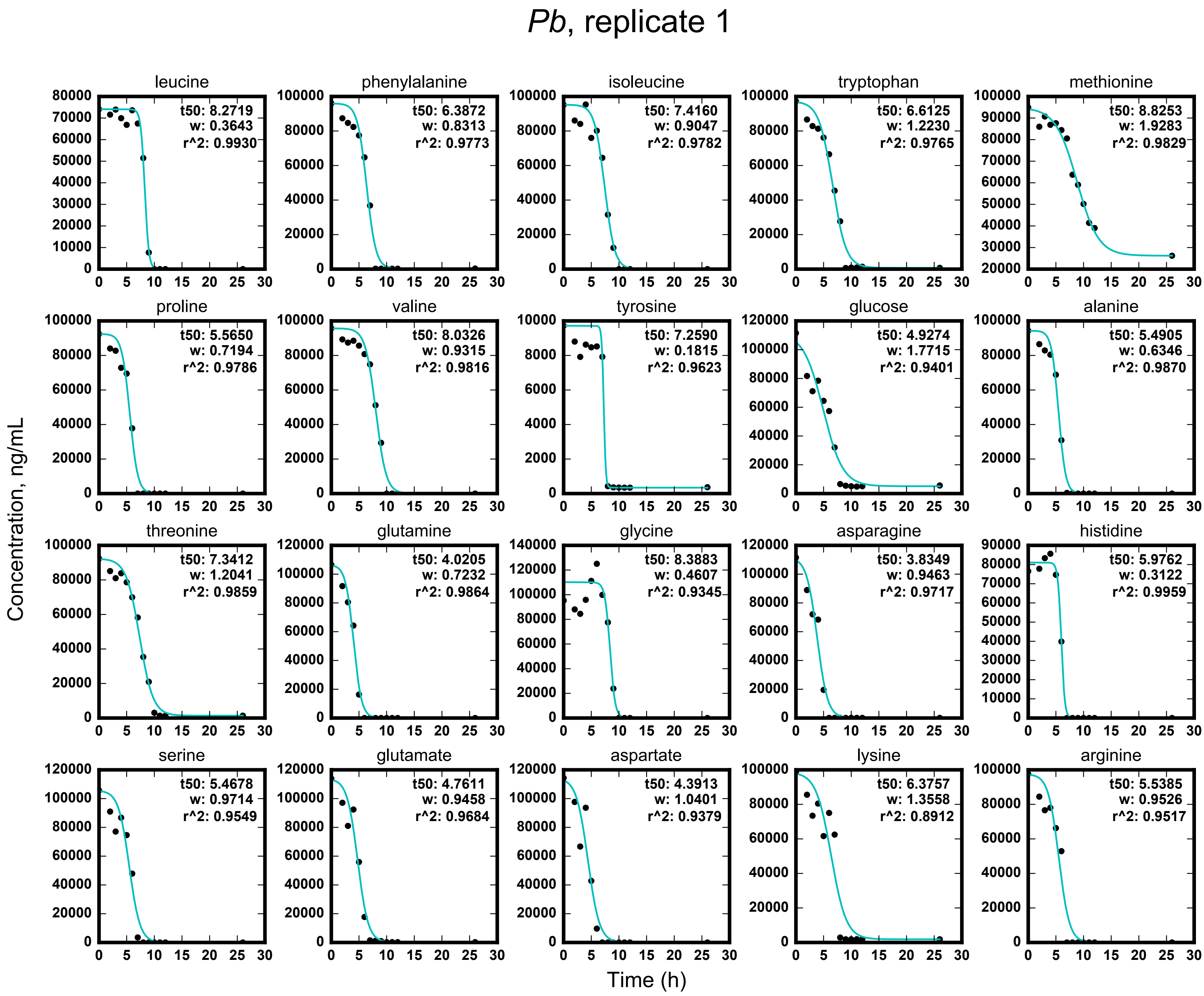
Pb, replicate 1
leucine
t50: 8.2719
w: 0.3643
r^2: 0.9930
phenylalanine
t50: 6.3872
w: 0.8313
r^2: 0.9773
isoleucine
t50: 7.4160
w: 0.9047
r^2: 0.9782
tryptophan
t50: 6.6125
w: 1.2230
r^2: 0.9765
methionine
t50: 8.8253
w: 1.9283
r^2: 0.9829
proline
t50: 5.5650
w: 0.7194
r^2: 0.9786
valine
t50: 8.0326
w: 0.9315
r^2: 0.9816
tyrosine
t50: 7.2590
w: 0.1815
r^2: 0.9623
glucose
t50: 4.9274
w: 1.7715
r^2: 0.9401
alanine
t50: 5.4905
w: 0.6346
r^2: 0.9870
threonine
t50: 7.3412
w: 1.2041
r^2: 0.9859
glutamine
t50: 4.0205
w: 0.7232
r^2: 0.9864
glycine
t50: 8.3883
w: 0.4607
r^2: 0.9345
asparagine
t50: 3.8349
w: 0.9463
r^2: 0.9717
histidine
t50: 5.9762
w: 0.3122
r^2: 0.9959
serine
t50: 5.4678
w: 0.9714
r^2: 0.9549
glutamate
t50: 4.7611
w: 0.9458
r^2: 0.9684
aspartate
t50: 4.3913
w: 1.0401
r^2: 0.9379
lysine
t50: 6.3757
w: 1.3558
r^2: 0.8912
arginine
t50: 5.5385
w: 0.9526
r^2: 0.9517
Concentration, ng/mL
Time (h)

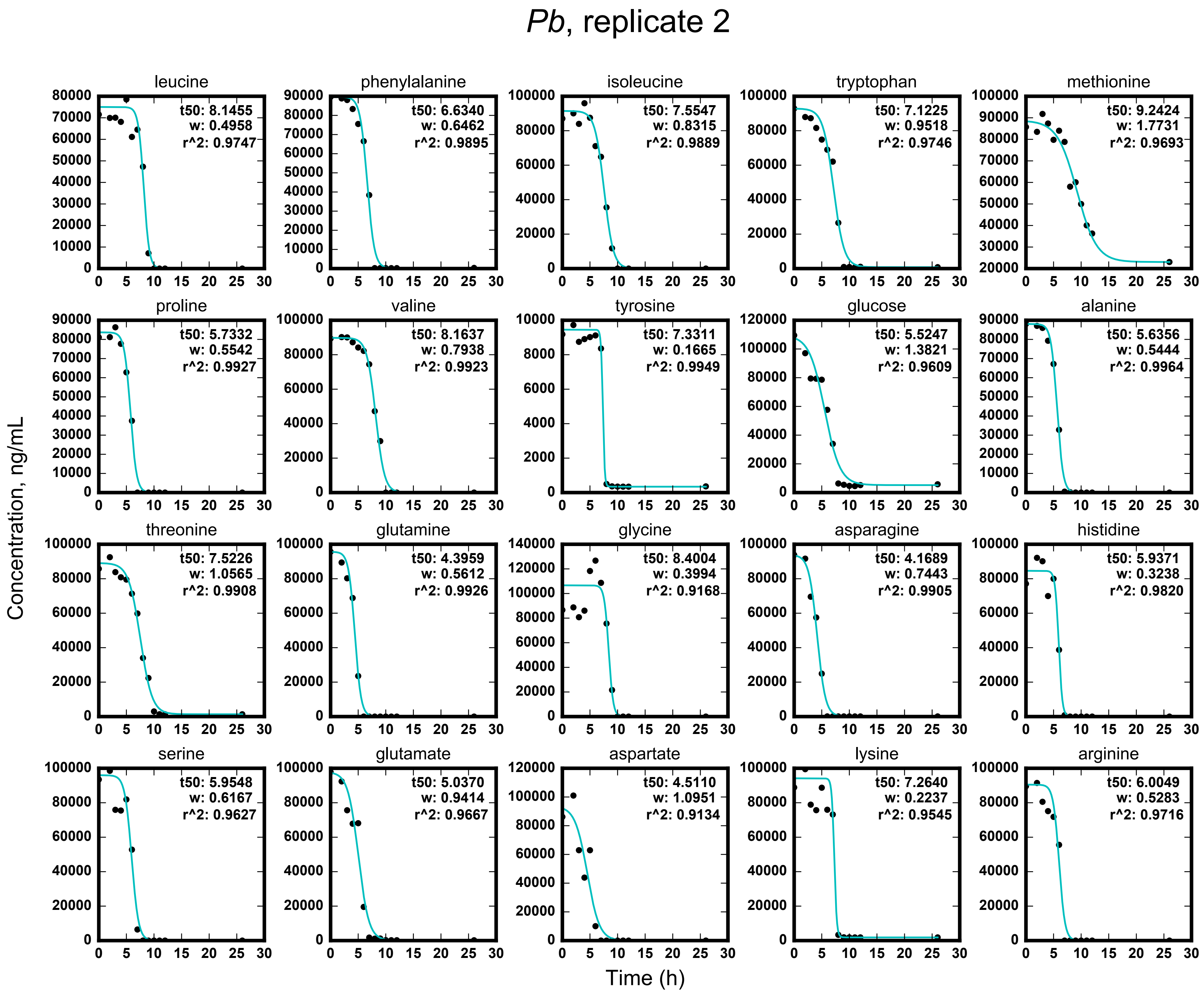
Pb, replicate 2
leucine
t50: 8.1455
w: 0.4958
r^2: 0.9747
phenylalanine
t50: 6.6340
w: 0.6462
r^2: 0.9895
isoleucine
t50: 7.5547
w: 0.8315
r^2: 0.9889
tryptophan
t50: 7.1225
w: 0.9518
r^2: 0.9746
methionine
t50: 9.2424
w: 1.7731
r^2: 0.9693
proline
t50: 5.7332
w: 0.5542
r^2: 0.9927
valine
t50: 8.1637
w: 0.7938
r^2: 0.9923
tyrosine
t50: 7.3311
w: 0.1665
r^2: 0.9949
glucose
t50: 5.5247
w: 1.3821
r^2: 0.9609
alanine
t50: 5.6356
w: 0.5444
r^2: 0.9964
threonine
t50: 7.5226
w: 1.0565
r^2: 0.9908
glutamine
t50: 4.3959
w: 0.5612
r^2: 0.9926
glycine
t50: 8.4004
w: 0.3994
r^2: 0.9168
asparagine
t50: 4.1689
w: 0.7443
r^2: 0.9905
histidine
t50: 5.9371
w: 0.3238
r^2: 0.9820
serine
t50: 5.9548
w: 0.6167
r^2: 0.9627
glutamate
t50: 5.0370
w: 0.9414
r^2: 0.9667
aspartate
t50: 4.5110
w: 1.0951
r^2: 0.9134
lysine
t50: 7.2640
w: 0.2237
r^2: 0.9545
arginine
t50: 6.0049
w: 0.5283
r^2: 0.9716
Concentration, ng/mL
Time (h)

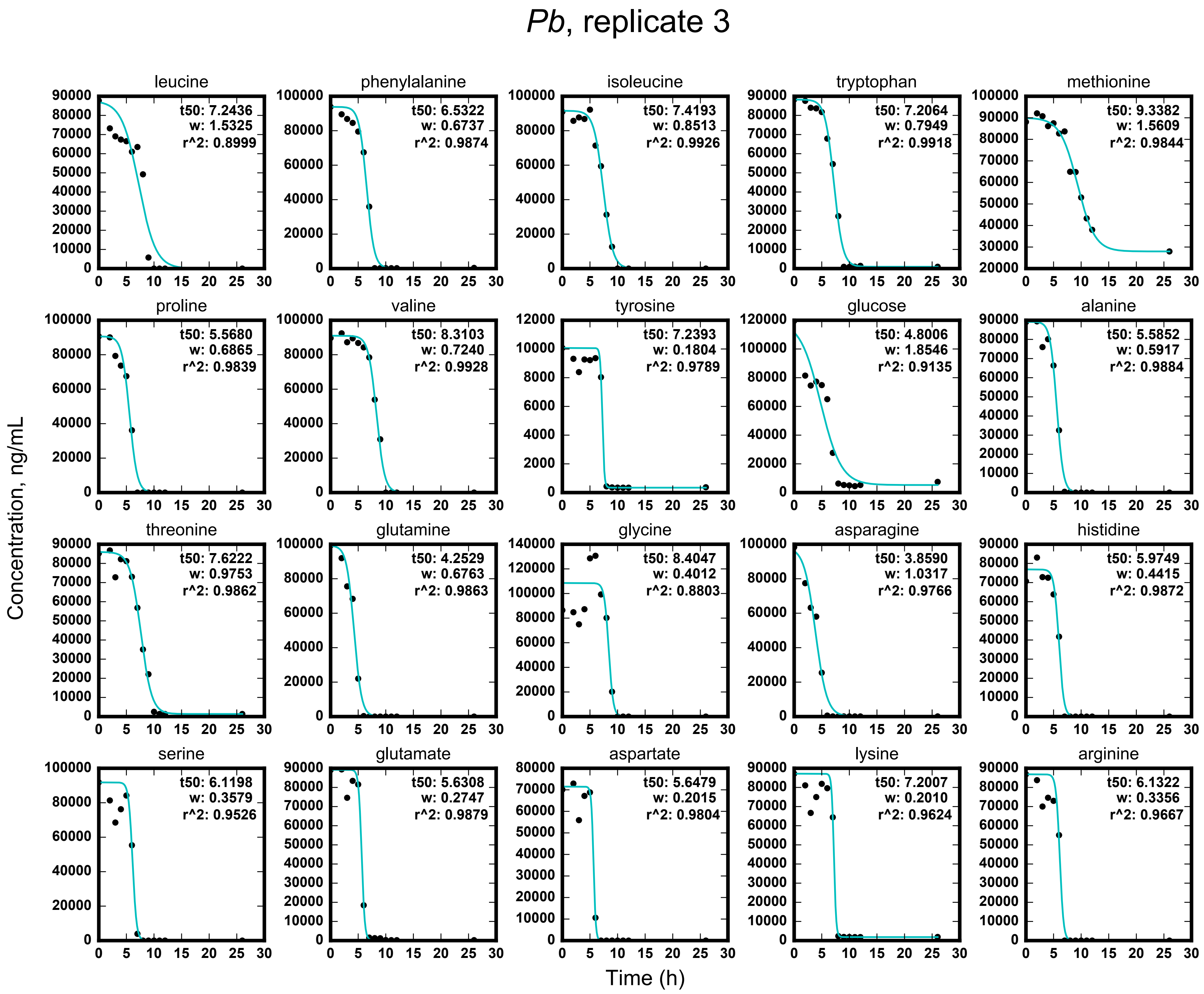

Pb, replicate 3
leucine
t50: 7.2436
w: 1.5325
r^2: 0.8999
phenylalanine
t50: 6.5322
w: 0.6737
r^2: 0.9874
isoleucine
t50: 7.4193
w: 0.8513
r^2: 0.9926
tryptophan
t50: 7.2064
w: 0.7949
r^2: 0.9918
methionine
t50: 9.3382
w: 1.5609
r^2: 0.9844
proline
t50: 5.5680
w: 0.6865
r^2: 0.9839
valine
t50: 8.3103
w: 0.7240
r^2: 0.9928
tyrosine
t50: 7.2393
w: 0.1804
r^2: 0.9789
glucose
t50: 4.8006
w: 1.8546
r^2: 0.9135
alanine
t50: 5.5852
w: 0.5917
r^2: 0.9884
threonine
t50: 7.6222
w: 0.9753
r^2: 0.9862
glutamine
t50: 4.2529
w: 0.6763
r^2: 0.9863
glycine
t50: 8.4047
w: 0.4012
r^2: 0.8803
asparagine
t50: 3.8590
w: 1.0317
r^2: 0.9766
histidine
t50: 5.9749
w: 0.4415
r^2: 0.9872
serine
t50: 6.1198
w: 0.3579
r^2: 0.9526
glutamate
t50: 5.6308
w: 0.2747
r^2: 0.9879
aspartate
t50: 5.6479
w: 0.2015
r^2: 0.9804
lysine
t50: 7.2007
w: 0.2010
r^2: 0.9624
arginine
t50: 6.1322
w: 0.3356
r^2: 0.9667
Concentration, ng/mL
Time (h)
